# Recurrent, Complicated Diverticulitis With Atypical Features

**DOI:** 10.7759/cureus.17983

**Published:** 2021-09-15

**Authors:** Shelley Persaud, Bir Singh, Francisco Brea, Johnathan Frunzi

**Affiliations:** 1 Internal Medicine, Medical Center of Trinity, Trinity, USA

**Keywords:** sigmoid diverticulitis, complicated diverticulitis, diverticulosis, diverticular abscess, diverticulitis recommendations

## Abstract

Diverticular disease is a common condition responsible for significant costs to the healthcare system in the Western world. It ranges from asymptomatic diverticulosis to complicated diverticulitis. Here, we present a unique case of recurrent, complicated diverticulitis in a 62-year-old Caucasian male. Within a span of one year, he was hospitalized six times with diverticulitis before undergoing elective sigmoid colon resection. Imaging showed diverticulitis of distal descending and proximal sigmoid colon with sealed perforation, recurrent abscesses, and formation of colocutaneous fistulas. During each hospitalization, the patient was advised to follow up with general surgery and/or outpatient gastroenterology but chose not to do so. Eventually, he required an elective sigmoid colectomy with a takedown of the colocutaneous fistulas. In this case report, we discuss the atypical features and criteria for prophylactic colon resection in diverticulitis to highlight the importance of outpatient follow-up with general surgery and gastroenterology.

## Introduction

Diverticular disease of the colon is a prevalent gastrointestinal disorder in the Western world that is associated with significant morbidity and healthcare costs. More than 50% of Americans older than 60 years of age have diverticular disease. Annual outpatient visits and hospital admissions account for more than $2 billion in costs [1]. Risk factors include red meat consumption, obesity, smoking, and a sedentary lifestyle. The pathophysiology of diverticular disease is complex and poorly understood. A prevailing hypothesis is that the complex interaction between genetics, diet, lifestyle, and medications (i.e., nonsteroidal anti-inflammatory drugs) alters the gut microbiome. This, in turn, causes defects in the mucosal barrier and immune function triggering an inflammatory cascade and mucosal inflammation. High-fiber diets, however, increase microbiome diversity, which increases the production of mucus and antimicrobial peptides that mediate immune homeostasis and promote intestinal barrier function [1].

In contrast to asymptomatic diverticulosis, diverticulitis usually causes left lower quadrant abdominal pain, especially in the Western world [2]. Interestingly, in Asia, it is more common to see diverticulitis presenting as right lower quadrant pain; patients tend to be younger with more right-sided colon involvement likely due to congenital reasons [2]. Diagnosis is made with a combination of clinical findings and imaging. Imaging evaluates for complications such as fistula, abscess, bowel obstruction, or perforation [3]. According to the American College of Gastroenterology (ACG), antibiotics should be selectively used in acute, uncomplicated diverticulitis but withheld in mild cases. ACG recommends against elective colon resection in patients with an initial, uncomplicated episode of acute diverticulitis. The decision to perform elective, prophylactic colon resection should be individualized.

## Case presentation

**Figure 1:**
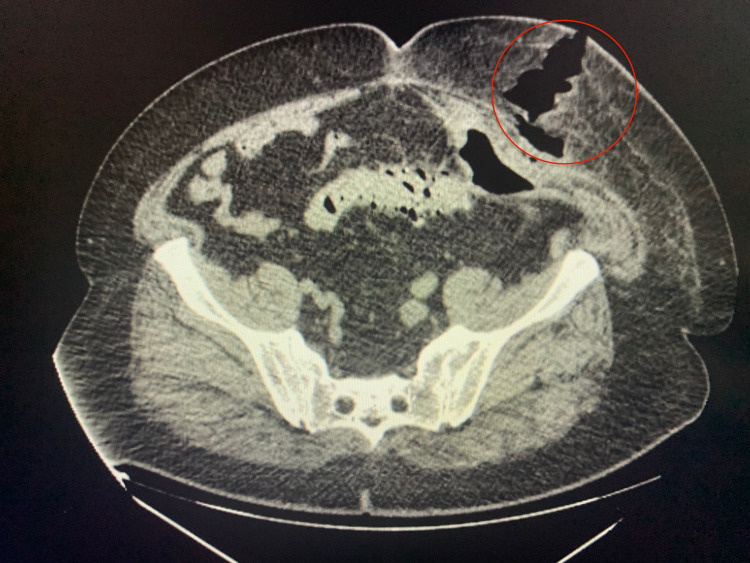
Thickened portion of L rectus abdominis muscle associated with an extensive amount of intramuscular and subcutaneous gas (red circle) compatible with enterocutaneous fistula.

**Figure 2:**
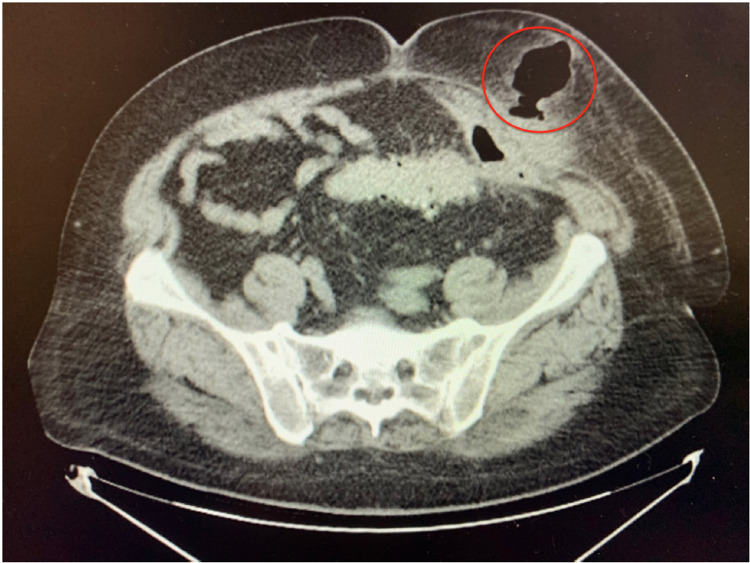
Persistent enterocutaneous fistula and increased subcutaneous emphysema (delineated by the red circle).

A 62-year-old Caucasian male with a medical history of chronic kidney disease stage III and hypertension presented with severe left lower quadrant pain for three days. Imaging showed acute diverticulitis involving the junction of distal descending and proximal sigmoid colon with sealed perforation and diverticular abscess. He was started on ciprofloxacin and metronidazole and underwent computed tomography (CT)-guided drainage of the left lower quadrant abscess. His abdominal pain improved, and he was discharged with instructions to follow up with gastroenterology for colonoscopy in six to eight weeks. The patient chose not to follow up and presented four months later with another episode of diverticulitis. CT of the abdomen and pelvis showed an extensive amount of intramuscular and subcutaneous gas compatible with enterocutaneous fistula, as seen in Figure 1. He was treated with antibiotics and advised to have a colonoscopy after resolution of diverticulitis and elective sigmoid colon resection. Two months later, the patient was readmitted for the third time and found to have persistent enterocutaneous fistula and increased subcutaneous emphysema, as seen in Figure 2.

The patient required percutaneous drain placement and intravenous antibiotics. One month later, he was readmitted for the fourth time with drainage from his abscess at the site of the colocutaneous fistula, requiring a colostomy bag. Inpatient gastroenterology service was consulted and a colonoscopy was performed, which showed diverticulosis, mild localized, inflammatory change in the sigmoid colon, with small internal hemorrhoids. One month later, he received an elective sigmoid colon resection with a takedown of colocutaneous fistulas.

## Discussion

This patient’s presentation with recurrent diverticulitis has many atypical features including the recurrence and the complicated nature of the first presentation of diverticulitis. Though recurrent episodes of acute diverticulitis are seen in 20-35% of patients, a study examining 672 patients showed that less than 4% developed complicated diverticular disease with fistula, abscess, or frank perforation [3]. Within a span of nine months, our patient was admitted on at least five occasions with complicated diverticulitis, including a sealed perforation, colocutaneous fistula, and multiple abscesses. Predictors of recurrence included retroperitoneal abscess, family history, and left-sided colonic involvement [4]. Aside from the left-sided colonic involvement, our patient did not meet other criteria. Generally, studies have demonstrated that recurrent episodes of diverticulitis do not present with further complications [5]. Our patient’s third and fourth admissions, however, demonstrated a colocutaneous fistula, which is seen in less than 5% of patients [6].

In another study examining 241 diverticulitis patients with an intra-abdominal abscess, only 4% had recurrent diverticular abscesses [7]. Our patient developed a diverticular abscess on four occasions, requiring percutaneous drainage and antibiotics. One was a polymicrobial abscess, requiring a six-week course of intravenous antibiotics with meropenem and linezolid. Approximately 67% of initial presentations of diverticulitis are uncomplicated, but this patient’s first admission was with a diverticular abscess and sealed perforation [8].

An important issue this case raises is when should diverticulitis be managed surgically. As mentioned, the ACG has no specific recommendations at this time. We feel that the severity of presentation should be a major criterion when considering surgical intervention. Because this patient’s first admission with diverticulitis was complicated and he developed a recurrence one month later, he was a candidate for elective colon resection.

## Conclusions

This case highlights the importance of outpatient gastroenterology and general surgery follow-up in the management of diverticulitis. During each hospitalization, our patient was advised to receive a colonoscopy six to eight weeks after discharge and follow up with surgery but did not do so. He reported having difficulty maintaining healthcare insurance. It is highly likely that the patient’s failure to comply played a major role in his recurrent hospitalizations for complicated diverticulitis.
